# Primary Pancreatic Hydatid: A Rare Cystic Lesion of the Pancreas

**DOI:** 10.4269/ajtmh.15-0713

**Published:** 2017-04-05

**Authors:** Sonali Sethi, Sunil Kumar Puri, Anil Agarwal

**Affiliations:** 1 Department of Radiology, Govind Ballabh Pant Hospital, New Delhi, India; 2 Department of Gastrointestinal Surgery, Govind Ballabh Pant Hospital, New Delhi, India

A 48-year-old woman presented with vague abdominal pain and malaise. She was from Andhra Pradesh, an area endemic for hydatid disease due to *Echinococcus granulosus*. The laboratory investigations were indicative of anemia with hemoglobin of 6 g/dL. Serum bilirubin and amylase were within normal limits and the abdominal examination was benign.

An ultrasound of the abdomen was performed which revealed an approximately 11 × 14-cm-sized well-defined cystic lesion with hyperechoic walls and internal echogenic floating serpentine structures arising from the tail of the pancreas (Figure 1

**Figure 1.**
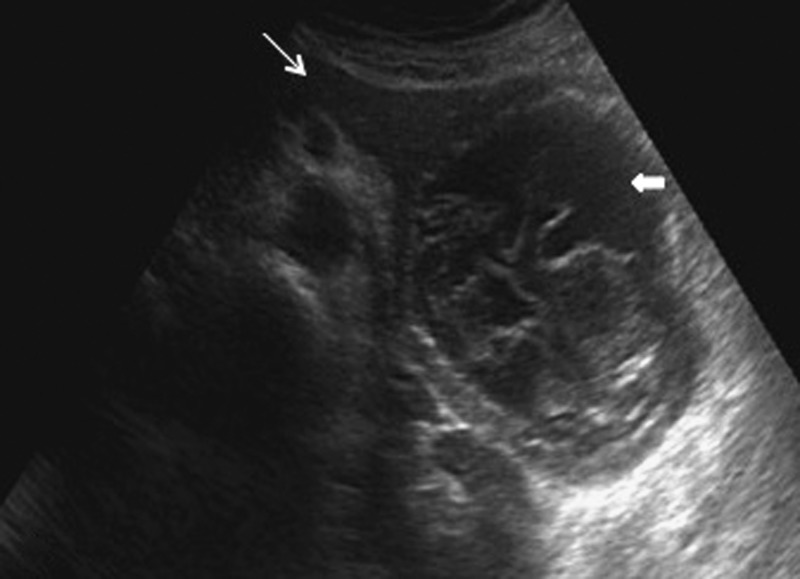
Ultrasound sonography image showing a well-defined anechoic lesion with thick double-lined hyperechoic wall and internal echogenic serpentine structures arising from the distal body and the tail of the pancreas (bold white arrow). The proximal body and the head of the pancreas appear normal (thin white arrow).

).

Further evaluation was done by performing a contrast-enhanced computed tomography scan through the abdomen using the pancreatic protocol. It revealed a unilocular cystic lesion in the body and the tail of the pancreas. The head of the pancreas showed normal homogenous enhancement and normal opacification of the retropancreatic splenic vein with few hyperdense foci in the internal contents (Figure 2

**Figure 2.**
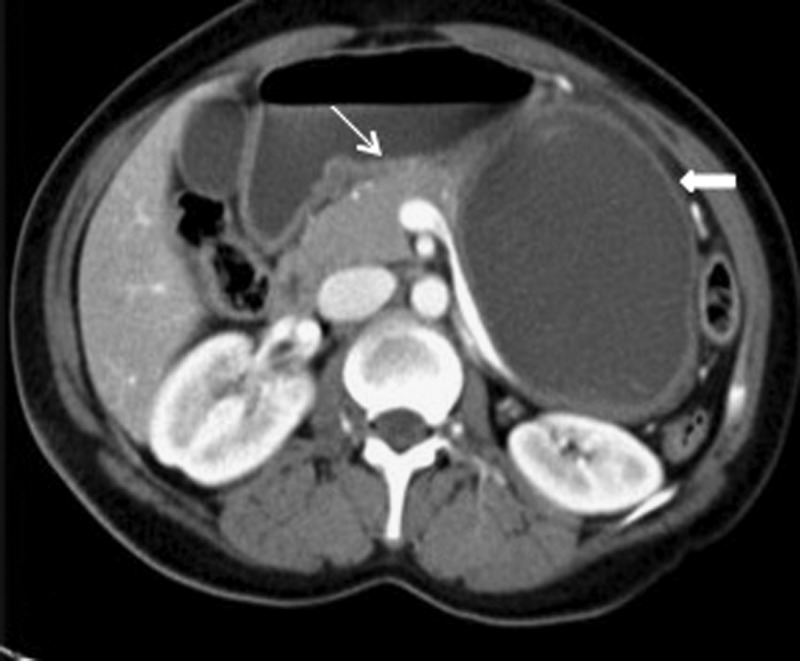
Multidetector computed tomography axial image showing a well-defined unilocular nonenhancing cystic structure (bold white arrow) arising from the distal body and the tail of the pancreas, with internal hyperdense foci. The normal enhancing pancreatic parenchyma is seen proximally (thin white arrow).

).

A magnetic resonance imaging of the abdomen revealed a well-defined T2W homogeneously hyperintense lesion in the body and the tail of the pancreas with a hypointense rim and internal serpiginous hypointense structures suggestive of detached membranes (Figure 3

**Figure 3.**
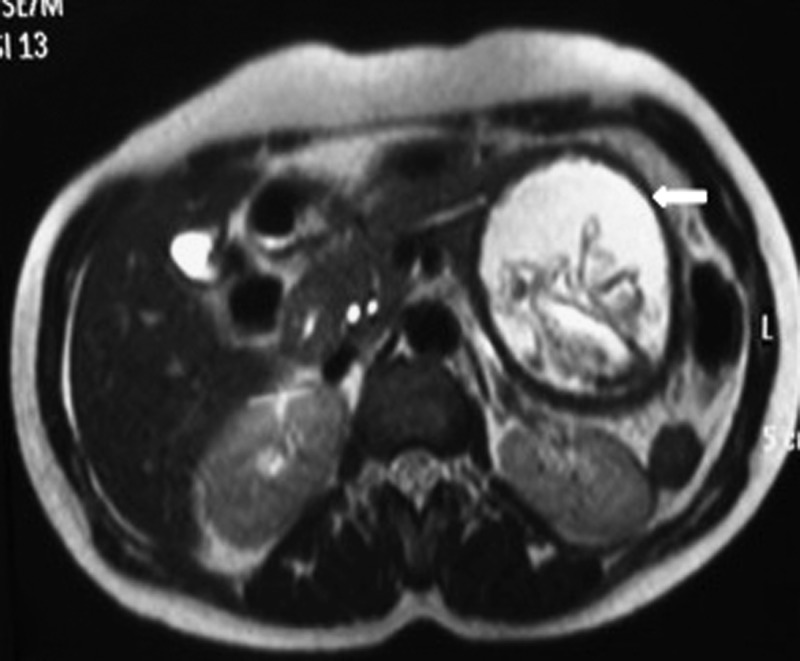
Magnetic resonance imaging abdomen axial T2-weighted image showing a well-defined altered signal intensity lesion with a thick complete hypointense rim and detached membranes from the distal body and tail of the pancreas (bold white arrow).

). A diagnosis of a hydatid cyst with detached membranes was made consistent with a CE3a stage according to the World Health Organization ultrasound classification.^1^

Intraoperatively, a 11 × 14–cm-sized cystic lesion was found arising from the distal pancreas (Figure 4

**Figure 4.**
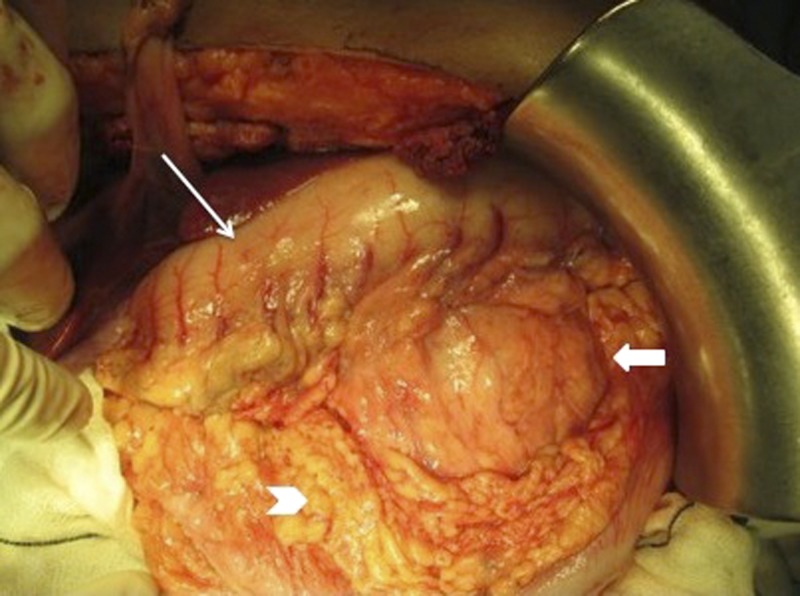
Intraoperative image with the pancreatic cystic lesion (bold white arrow). The stomach is seen anteriorly (thin white arrow) and the transverse mesocolon posteriorly (white arrow head).

). Postoperative histology was consistent with hydatid disease showing ectocyst and internal detached membranes (Figure 5

**Figure 5.**
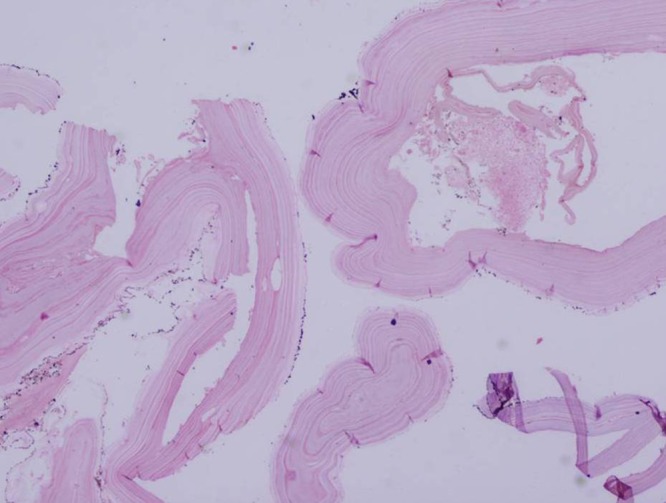
Histopathology image showing laminated membranes and protoscolices and hydatid sand.

). The fluid was positive for hydatid sand, scolices, and hooklets.

## Discussion

Hydatid disease is caused by the larval stage of *E. granulosus* and remains a public health problem in cattle-raising countries which are endemic for the disease. Man is an accidental intermediate host and dog is a definitive host. The highest prevalence of hydatid disease in humans in India has been reported from south Indian states.^2^ The commonest site of involvement is the liver, seen in 59–75% cases. Pulmonary, renal, skeletal, and central nervous system hydatidosis is less common.^3^ Pancreatic hydatid disease is extremely rare and accounts for 0.25% of the cases.^4^ The closest differential diagnoses are mucinous cystadenoma and pseudocyst of the pancreas. The treatment of choice is surgical resection in the form of a distal pancreatectomy for lesions of the distal pancreas and a cystopericystectomy for the head lesions. Pre- and postoperative treatment with a 1-month course of albendazole and a 2-week course of praziquantel is recommended.^1^,^5^
